# Genes Important for Catalase Activity in *Enterococcus faecalis*

**DOI:** 10.1371/journal.pone.0036725

**Published:** 2012-05-10

**Authors:** Michael Baureder, Lars Hederstedt

**Affiliations:** Microbiology Group, Department of Biology, Lund University, Lund, Sweden; University of Padova, Italy

## Abstract

Little in general is known about how heme proteins are assembled from their constituents in cells. The Gram-positive bacterium *Enterococcus faecalis* cannot synthesize heme and does not depend on it for growth. However, when supplied with heme in the growth medium the cells can synthesize two heme proteins; catalase (KatA) and cytochrome *bd* (CydAB). To identify novel factors important for catalase biogenesis libraries of *E. faecalis* gene insertion mutants were generated using two different types of transposons. The libraries of mutants were screened for clones deficient in catalase activity using a colony zymogram staining procedure. Analysis of obtained clones identified, in addition to *katA* (encoding the catalase enzyme protein), nine genes distributed over five different chromosomal loci. No factors with a dedicated essential role in catalase biogenesis or heme trafficking were revealed, but the results indicate the RNA degradosome (*srmB*, *rnjA*), an ABC-type oligopeptide transporter (*oppBC*), a two-component signal transducer (*etaR*), and NADH peroxidase (*npr*) as being important for expression of catalase activity in *E. faecalis*. It is demonstrated that catalase biogenesis in *E. faecalis* is independent of the CydABCD proteins and that a conserved proline residue in the N-terminal region of KatA is important for catalase assembly.

## Introduction

Catalase is a well-known enzyme found in all three domains of life. It plays a role in oxidative stress defense by degrading hydrogen peroxide to molecular oxygen and water [1].

*Enterococcus faecalis* is one of the few species of lactic acid bacteria that exhibit catalase activity, but only when grown in the presence of heme [2]. *E. faecalis* catalase (KatA) belongs to the group of heme-containing mono-functional catalases (EC 1.11.1.6). It is a homo-tetrameric protein containing one heme group (protoheme IX) per subunit and its crystal structure has been determined [3]. Catalase protects *E. faecalis* against hydrogen peroxide stress but is not the only enzyme with this function. The bacterium can produce a NADH peroxidase (Npr) that degrades hydrogen peroxide to water and which seems sufficient for protection against high concentrations of hydrogen peroxide when heme is not available [4]. *E. faecalis* cells cannot synthesize heme and thus have to take it up from the environment in order to form active catalase.

Despite the fact that catalase has been studied intensively for many years biogenesis of this enzyme is not understood. The chronological order of assembly events such as KatA polypeptide folding, tetramer formation and heme insertion into the apoprotein is not known. The complex structure of the catalase tetramer as well as the hydrophobic nature of heme and its potential in generating reactive oxygen species indicate the need for tightly controlled and assisted assembly of catalase. No catalase biogenesis factors have been identified except in the bacterium *Campylobacter jejuni* which was recently reported to contain a protein (Cj1386) involved in heme trafficking to catalase [5]. *E. faecalis* does not encode any apparent homolog of this protein.

In this work we used *E. faecalis* as an experimental system to search for genes encoding factors necessary for biogenesis of functional catalase, i.e. genes for proteins that function in heme uptake, heme trafficking or catalase assembly. We generated libraries of transposon insertion mutants in *E. faecalis* strain OG1RF which were screened for clones deficient in catalase activity. Identification of the chromosomal transposon insertion site in those mutants revealed a set of genes in addition to *katA*.

## Results

### Colony assay for detection of catalase activity

The classical method for detection of catalase activity in bacteria grown on agar plates is the observation of effervescence after flushing colonies on the plate with dilute hydrogen peroxide solution. With this procedure it is difficult to find rare catalase-deficient colonies among a large number of catalase-positive colonies. We therefore made use of a zymogram staining reagent, modified from that described by Hanker and Rabin [6], which leads to the formation of a purple color around colonies due to catalase activity.

**Figure 1:**
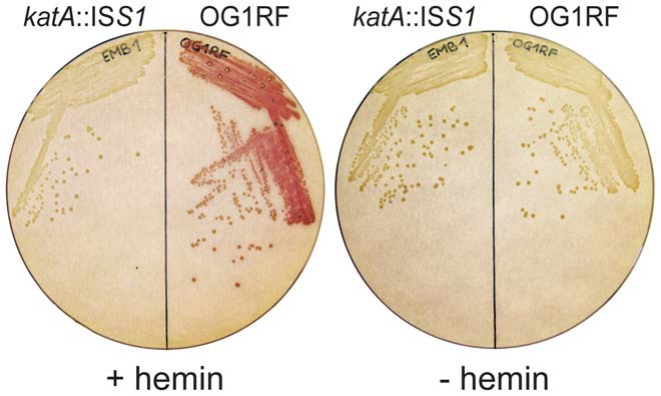
Colony catalase activity assay. *E. faecalis* OG1RF (wild-type) and EMB1 (*katA*::IS*S1*) colonies grown on TH agar plates with and without 8 µM hemin added and zymogram stained for catalase activity.

The applicability of this assay for *E. faecalis* was tested by staining colonies of strains OG1RF (wild-type) and EMB1 (*katA*::IS*S1*) grown on Todd Hewitt agar plates supplemented with hemin. The color reaction was observed for OG1RF while EMB1 remained unstained. As expected, colonies of these strains did not stain after growth on media without hemin (Figure 1).

### Transposon mutagenesis and screening of libraries

**Figure 2:**
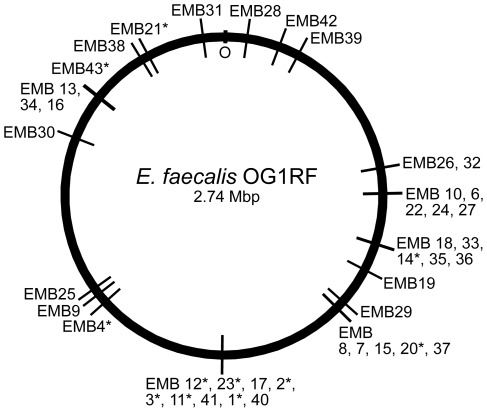
Distribution of transposon insertion sites. Transposon insertion sites in the OG1RF genome of 42 catalase-deficient mutants isolated in this work are shown. The strain names (EMBx) are indicated and IS*S1*-derived mutants are marked with an asterisk.

**Figure 3:**
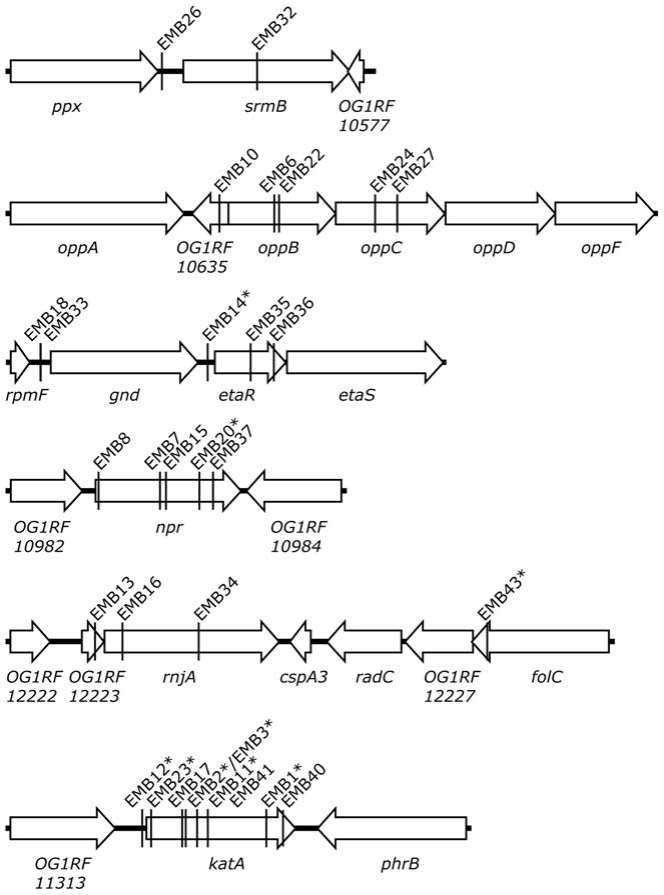
Genomic context of loci for which two or more independent catalase-deficient transposon insertion mutants were obtained. Insertion sites in the *E. faecalis* OG1RF chromosome are marked with the designation of the respective isolate (EMBx) and IS*S1*-derived mutants are marked with an asterisk. See Table 1.

Two different transposon mutagenesis systems, pG^+^host8:IS*S1* [7] and EfaMarTn [8], were used to generate libraries of insertion mutants in *E. faecalis* OG1RF. The colony staining method described above was used to screen the libraries for mutants lacking catalase activity when grown on agar plates containing hemin. In total 8,180 IS*S1* and 7,994 EfaMarTn transposon insertion mutants were tested for catalase activity resulting in the isolation of 11 IS*S1* and 32 EfaMarTn mutants with no or reduced catalase activity stain. The transposon insertion site in the chromosome of 42 of these mutants was identified by inverse PCR combined with DNA sequence analysis. The insertion site in one strain, EMB5, could not be identified. All isolated catalase-deficient clones seemingly contained a single transposon insertion, i.e. amplification of Tn insertion sites by inverse PCR yielded only one product per clone. The insertion sites were found distributed over 23 different loci on the OG1RF chromosome (Figure 2, Tables 1 and S2). For six loci two or more independent transposon insertion mutants were obtained (Table 1 and Figure 3). One of them was the expected OG1RF_11314 (*katA*) gene which validated the used mutagenesis and screening procedure for isolation of catalase-deficient mutants. The other five loci were OG1RF_10576 (*srmB*), OG1RF_10635-37 (*opp*), OG1RF_10782-83 (*gnd* and *etaR*), OG1RF_10983 (*npr*), and OG1RF_12223-24 (*rnjA*).

**Table 1:** Loci for which two or more independent catalase-deficient transposon insertion mutants were obtained.

	Locus tag(s)^a^	Gene	Annotated function	Strain	Tn insertion position in genome^a^	Catalase activity^b^	KatA protein
**Locus 1**	OG1RF_10576	*srmB*	ATP-dependent RNA helicase DeaD	EMB26	604,149	0.10	*nd*
	OG1RF_10576			EMB32	605,068	0.33	0.37
**Locus 2**	OG1RF_10635	----	Hypothetical protein	EMB10	674,142	0.11	0.37
	OG1RF_10636	*oppB*	Oligopeptide ABC superfamily ATP binding cassette transporter, membrane protein	EMB6	674,670	0.12	0.30
	OG1RF_10636			EMB22	674,715	0.29	0.32
	OG1RF_10637	*oppC*	Oligopeptide ABC superfamily ATP binding cassette transporter, membrane protein	EMB24	675,641	0.42	0.35
	OG1RF_10637			EMB27	675,858	0.54	0.44
**Locus 3**	OG1RF_10782	*gnd*	Phosphogluconate dehydrogenase (decarboxylating)	EMB18	814,391	0.11	0.23
	OG1RF_10782			EMB33	814,391	0.25	0.24
	OG1RF_10783	*etaR*	Response regulator	EMB14^c^	815,997	0.19	0.26
	OG1RF_10783			EMB35	816,411	0.26	0.28
	OG1RF_10783			EMB36	816,640	0.29	0.34
**Locus 4**	OG1RF_10983	*npr*	NADH peroxidase	EMB8	1,022,177	0.08	0.90
	OG1RF_10983			EMB7	1,022,772	0.08	0.68
	OG1RF_10983			EMB15	1,022,828	0.15	1.00
	OG1RF_10983			EMB20^c^	1,023,154	0.05	0.96
	OG1RF_10983			EMB37	1,023,286	0.12	1.19
**Locus 5**	OG1RF_12223	----	Hypothetical protein	EMB16	2,343,191	0.42	0.83
	OG1RF_12224	*rnjA*	Ribonuclease J1	EMB13	2,343,456	0.61	0.94
	OG1RF_12224			EMB34	2,344,190	0.98	1.12
**Locus 6**	OG1RF_11314	*katA*	Catalase	EMB12^c^	1,371,597	*na*	*na*
	OG1RF_11314			EMB23^c^	1,371,681		
	OG1RF_11314			EMB17	1,371,981		
	OG1RF_11314			EMB2^c^	1,372,016		
	OG1RF_11314			EMB3^c^	1,372,016		
	OG1RF_11314			EMB11^c^	1,372,126		
	OG1RF_11314			EMB41	1,372,226		
	OG1RF_11314			EMB1^c^	1,372,790		
	OG1RF_11314			EMB40	1,372,951		

In the screen for catalase deficiency two or more independent transposon insertions were obtained in six loci. Catalase activity and KatA content in cell extracts are expressed as fraction relative to those of the parental strain OG1RF.

*nd*, not done; *na*, not applicable.

a GenBank: CP002621.1.

b Catalase activity of strain OG1RF was 14 U/mg of protein.

c IS*S1* system.

The isolation of several independent insertions in some genes indicated that the screen was near saturation and strongly suggested linkage between genetic locus and selected phenotype. Transposon insertions often generate polar effects on downstream genes. For this reason we cannot conclude whether the respective observed phenotype is caused by the gene disruption or a polar effect or a combination of the two. Insertions in three genes (*katA*, *npr*, and *etaR*) were obtained with both transposon mutagenesis systems.

### Catalase activity and KatA polypeptide in cell lysates

The catalase-deficient phenotype of the mutants found in the screen was first confirmed by catalase activity determination and immunoblot analysis for KatA polypeptide in the soluble (cytoplasmic) fraction of cell extracts. Subsequently unfractionated cell lysates of the mutants were similarly analyzed for catalase. Extracts of *E. faecalis* OG1RF served as reference in the analyses. The results are presented in Tables 1 and S2.

Catalase activity in the extracts correlated with the KatA polypeptide concentration except for the five *npr* insertion mutants which showed wild-type levels of the enzyme protein but only low activity. Notably, *rnjA* insertion mutants uniquely showed 5–10 fold decreased catalase activity when the cells were grown in BHI supplemented with hemin compared to TSBG supplemented with hemin. Irrespective of growth medium the colony size of these mutants was reduced compared to the wild-type and other catalase-deficient mutants.

### Identification of a *cydC katA* double mutant

To exclude the possibility of fortuitous mutations in *katA* in transposon insertion mutants the *katA* gene of mutants showing no catalase activity stain was sequenced. A single strain, EMB4 with *cydC* (the OG1RF_11664 gene) inactivated by transposon insertion (Tables 2 and S2), was found to carry a point mutation (C82A) in *katA* which results in a threonine instead of a proline residue at position 28 in KatA. Full-length KatA polypeptide was found in the particulate cell fraction of strain EMB4. This is in contrast to the parental strain OG1RF and all other KatA-containing mutants which had the polypeptide in the soluble fraction. Cell lysates, as well as the particulate and soluble fractions, of EMB4 lacked catalase activity (<5% activity compared to OG1RF).

**Table 2:** Bacterial strains.

	Strain	Characteristics	Reference/source
***Enterococcus faecalis***	OG1RF	Rifampicin and fusidic acid resistant	Laboratory stock
	OG1RF/pCJK55	EfaMarTn recipient	[8]
	CK111/pCF10-101/pCJK72	EfaMarTn donor	[8]
	OG1RF/pG^+^host8:IS*S1*	IS*S1* system	[7] & this work
	EMB4	*cydC*::IS*S1*, *katA82*	This work
	EMB44	Δ*cydABCD*::*tetL*	This work
***Escherichia coli***	TOP10	Cloning host	Invitrogen
	TOP10/pLUMB25	*cydA'*-*tetL*-*cydD'* in pCR-Blunt II TOPO	This work
	TOP10/pLUMB28	*cydA'*-*tetL*-*cydD'* in pJRS233	This work

### Cytochrome *bd* is not important for catalase biogenesis

**Figure 4:**
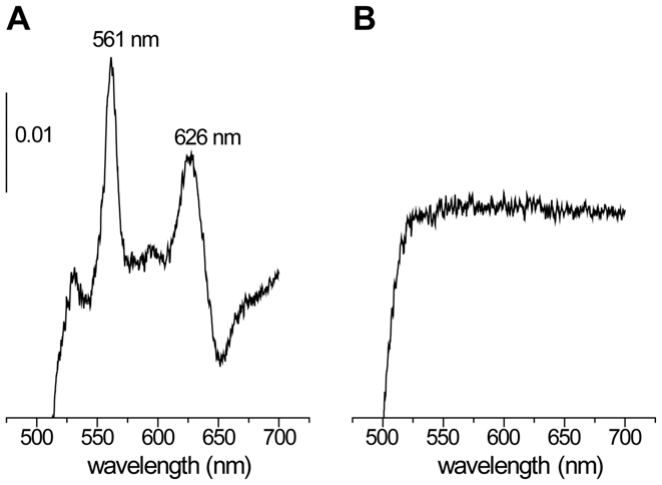
Light absorption spectra. Difference (dithionite-reduced minus air-oxidized) absorption spectra of cytoplasmic membranes isolated from strain OG1RF (A) and EMB4 (B) grown in the presence of hemin. The spectrum of strain EMB44 was similar to that of EMB4. The absorbance scale is indicated by the vertical bar.

Cytochrome *bd* and catalase are the only known heme proteins in *E. faecalis* [9]. Transposon insertion mutants defective in heme uptake or intracellular heme transport would show both cytochrome *bd* and catalase deficiency when grown in the presence of hemin. Light absorption redox spectroscopy of membranes isolated from cells grown in the presence of hemin demonstrated the presence of cytochrome *bd* in all isolated catalase-deficient mutants except for EMB4 (Figure 4). This property of strain EMB4 was expected since *cydC* and *cydD* of the *cydABCD* operon are in other bacteria known to be essential for the synthesis of cytochrome *bd* [10], [11], [12]. To analyze whether any of the CydABCD proteins play a role in heme trafficking or maturation of catalase the *cydABCD* operon was deleted in OG1RF, yielding strain EMB44. When grown in the presence of hemin this mutant contained normal amounts of both KatA polypeptide and catalase activity, and, as expected, lacked cytochrome *bd*. From these results we conclude that CydC is important for cytochrome *bd* biogenesis also in *E. faecalis* and that the CydABCD proteins are not important for biogenesis of catalase. The results furthermore show that the *katA82* point mutation alone is responsible for the catalase-negative phenotype of strain EMB4.

## Discussion

Biogenesis of catalase requires insertion of one heme group per KatA monomer and assembly of the homotetramer. How and in what order these events occur are not known. To identify genes important for biogenesis of a functional catalase enzyme and for heme trafficking, libraries of *E. faecalis* mutants were constructed using two different transposon systems and screened for clones defective in catalase activity.

**Figure 5:**
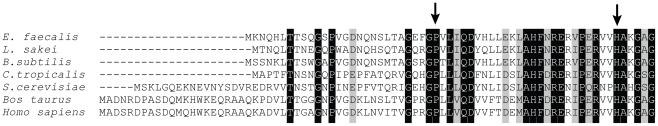
Sequence alignment of catalase polypeptides. Multiple amino acid sequence alignment of six catalases showing conserved residues in the N-terminal arm. Human and bovine catalase enzymes have an extended N-terminal region containing an additional α-helix. Residues Pro28 (mutated to a Thr in strain EMB4) and the essential His54 (proximal to heme iron) in *E. faecalis* KatA are indicated by arrows. *E. faecalis* (UniProt F2MTL2), *B. subtilis* (P26901), *Bos taurus* (P00432), *Homo sapiens* (P04040), *Lactobacillus sakei* (P30265), *Saccharomyces cerevisiae* (P15202), *Candida tropicalis* (P07820).

The multiple obtained *katA*-deficient mutants validated the used mutagenesis and screening procedure for isolation of catalase-deficient mutants. A *katA* point mutation (*katA82*) was detected in strain EMB4 carrying IS*S1* inserted in *cydC*. It is remarkable that EMB4 is defective in both of the only two known heme proteins of *E. faecalis*. Our analysis of mutants defective in catalase or cytochrome *bd* did not reveal any interdependence of biogenesis or activity of the two enzymes. KatA82 polypeptide, with Pro28 replaced by Thr, was found inactive in the particulate fraction of cell extracts. The mutated Pro residue is conserved in mono-functional catalases and present in the N-terminal arm which interconnects with a neighboring subunit in the fully assembled tetrameric catalase (Figure 5) [3]. Apparently residue Pro28 is important for assembly of functional catalase. We conclude that the mutant KatA protein aggregates and therefore is found in the particulate cell fraction.

In addition to *katA* we identified five chromosomal loci seemingly important for expression of catalase activity in *E. faecalis* cells. The proteins encoded by those genes have diverse functions such as global regulation of RNA turnover (*rnjA*, *srmB*), NADH oxidation and hydrogen peroxide detoxification (*npr*), stress response (*etaR*), and membrane transport (*oppBC*). The connection between these physiological functions and expression of catalase activity in colonies remains to be elucidated but are probably indirect as discussed here below.

Five catalase-deficient insertion mutants had the gene for NADH peroxidase (Npr) inactivated. Similar to catalase Npr degrades hydrogen peroxide but in contrast it oxidizes NADH and generates water (instead of molecular oxygen) [13]. Interestingly, the amount of KatA polypeptide in Npr-negative mutants was found to be normal but the catalase activity was low. Since catalase polypeptide is unstable without the heme cofactor inserted [2] the low activity seems not explained by lack of heme in assembled catalase. The connection between Npr-deficiency and low catalase activity remains unexplained.

Several mutants isolated in the screen for catalase deficiency carried the transposon insertion in a gene related to RNA degradation. Two mutants were found defective in ribonuclease J1 (*rnjA*) and one in a hypothetical protein encoded by a gene upstream of *rnjA* (Figure 3). Ribonuclease J1 plays a central role in mRNA turnover thereby indirectly influencing the expression of a multitude of genes (reviewed in [14]). It forms a complex with ribonuclease J2 (*rnjB*) for which a single mutant (EMB29; Table S2) was obtained in our screen. RNases J1/J2 are essential for growth of group A streptococci [15] and RNase J1 is essential in *Bacillus subtilis* [16], [17]. Our results indicate that neither J1 nor J2 is essential in *E. faecalis*. Recently, it was reported that J2 is not essential for *E. faecalis* and involved in regulation of Ebp pili expression [18]. The DEAD-box RNA helicase SrmB, for which two insertion mutants were found in our screen, is thought to be a part of the RNA degradosome. In *S. aureus*, the homologous RNA helicase, CshA, interacts with RNase J1 [19]. These findings together suggest that normal catalase synthesis depends on a fully functional RNA degradosome complex.

Three transposon insertion mutants were obtained for *etaR* encoding the response regulator of the EtaRS two-component system. This system shows high similarity to LisRK of *Listeria monocytogenes* [20] and CsrRS of *Streptococcus pyogenes* [21]. EtaRS is involved in stress response and virulence [22], [23]. Catalase deficiency in EtaRS-negative strains has not been reported, but this might have passed undetected in previous studies if hemin was not included in the growth media. The gene upstream of *etaR* encodes a putative 6-phoshogluconate dehydrogenase (Gnd). Two mutants with the same transposon insertion site in the *gnd* promoter region were obtained in our screen. Except for colocalization of the genes no connection between Gnd and EtaRS is apparent. The *lisRK* and *gnd* genes in *L. monocytogenes*, but not in *S. pyogenes*, have the same organization as in *E. faecalis*.

It is not known how heme is transported into *E. faecalis*. Five transposon insertions were found in *oppB*, *oppC* and a putative gene upstream of *oppB* (Figure 3) encoding an oligopeptide ABC transporter (OppBCDF). For unknown reasons no insertion was found in *oppD* or *oppF*. In *Escherichia coli*, the housekeeping dipeptide permease (DppBCDF) functions as a heme transporter [24] and heme is delivered to the permease by the periplasmic peptide-binding proteins DppA and MppA. It has been suggested that DppB and DppC bind heme and thus contribute to substrate specificity of the transporter. Two homologs of *E. coli* DppBCDF can be identified by a BLAST search of *E. faecalis* strain OG1RF; OppBCDF and OG1RF_12367-70. This could suggest that *E. faecalis* OppBCDF plays a role in heme uptake. To test this we grew strain EMB6, and the wild-type strain as a reference, in the presence of 0.1 µM hemin, which is a limiting concentration with respect to assembly of active catalase. No difference in amount of catalase between the strains was observed. This result suggests that *E. faecalis* OppBCDF does not transport heme or that it is a low affinity heme transporter present alongside other transporters with similar or higher affinity for heme.

The presence of cytochrome *bd* in all the isolated catalase-deficient mutants, except for EMB4 with the *cydC* gene inactivated, indicates that mutants with a general block in heme uptake or heme protein assembly cannot be found by the experimental approach taken in this work. Reasons for this can be (i) presence of functionally redundant proteins, (ii) bias in insertion sites for the transposons used for mutagenesis such that certain genes will not be mutated or be overrepresented, or (iii) that the sought function is essential for growth. There is also the possibility that catalase biogenesis in *E. faecalis* is not assisted by any specific protein. It is not known if the heme groups for catalase in the cytoplasm and cytochrome *bd* in the membrane are taken up and delivered by the same cellular route. The *cydCD* genes encode an ABC transporter which is essential for biosynthesis of cytochrome *bd* and might play a role in heme insertion into the oxidase (CydAB) specifically [10], [11]. The *cydABCD* operon is however not required for catalase biogenesis in *E. faecalis*, as demonstrated by the properties of a deletion mutant EMB44.

In *Streptococcus agalactiae*, alkyl hydroperoxide reductase C (AhpC) was shown to bind intracellular heme and thereby protects it from degradation [25]. In addition, it was recently found that intracellular heme homeostasis is controlled by heme sensing and efflux systems in *Lactococcus lactis* [26] and *S. agalactiae* [27]. Disruption of the two *S. agalactiae* efflux systems, PefAB and PefCD, led to accumulation of heme in the cell. In our screen for catalase-deficient mutants we did not reveal any putative heme efflux mutants, i.e., all ∼16,000 transposon insertion mutants grew in the presence of 8 µM hemin.

## Materials and Methods

### Bacterial strains and culture media

*E. faecalis* strains were grown in Todd-Hewitt (TH), Brain Heart Infusion (BHI) or Tryptic Soy Broth (TSB). The latter was supplemented with 1% glucose (TSBG). When indicated 8 µM hemin (Sigma-Aldrich) was added to the media from a 10 mM stock solution in dimethyl sulfoxide (DMSO).

### Colony catalase activity assay

The staining reagent [6] was prepared by mixing one part of 80 mg/mL dopamine (Fluka) in 0.2 M potassium phosphate buffer pH 8.0, one part of 40 mg/mL *p*-phenylenediamine (ICN Pharmaceuticals) in buffer, one part 12% hydrogen peroxide, and two parts of DMSO. Two mL of the reagent were mixed with 4 mL molten soft agar (0.7%) in water and subsequently poured on bacterial growth on an agar plate (d = 8.5 cm). Catalase activity caused within minutes the appearance of a purple color around bacterial colonies.

### Transposon mutagenesis – IS*S1* system

*E. faecalis* OG1RF was transformed with plasmid pG^+^host8:IS*S1* by electroporation as described in [28], but cells were incubated for 2 hours at 28°C after electroshock. Transformants were selected on TH plates containing 10 µg/mL tetracycline incubated at 28°C and the presence of the intact plasmid was checked by restriction enzyme digestion of isolated plasmid DNA with EcoRI and HindIII (New England Biolabs). Generation of random transposon mutants was done as described by Maguin *et al.* [7]; TH broth was inoculated with cells of strain OG1RF/pG^+^host8:IS*S1* to an OD_600_ of 0.05 and incubated for 2.5 hours at 28°C and 200 rpm. Then the culture was shifted to 37.5°C and incubated for a further 2.5 hours. Finally cells were diluted 1000-fold in 2 mM EDTA pH 7.5 and plated on TH agar containing 10 µg/mL tetracycline. After overnight incubation at 37°C transposants were picked and streaked on both TH containing 10 µg/mL tetracycline and TH containing additionally 8 µM hemin (100 mutants per plate). Colonies on the latter plate were stained for catalase activity.

### Transposon mutagenesis – EfaMarTn system

Generation of random transposon mutants was done as described by Kristich *et al.* [8]. In short; strains *E. faecalis* CK111/pCF10-101/pCJK72 (donor) and *E. faecalis* OG1RF/pCJK55 (recipient) were grown in BHI broth containing 10 µg/mL erythromycin. After overnight incubation the cultures were diluted 20-fold in BHI containing 1 µg/mL erythromycin. Nisin (Sigma-Aldrich) was added to the recipient culture to a final concentration of 10, 20, 30 or 40 ng/mL and the cultures were incubated at 30°C for 105 minutes. Donor and recipient were then mixed, plated on BHI containing the same concentration of nisin, and incubated at 30°C for 21 hours. All cells on the plate were suspended in BHI containing 2 mM EDTA pH 7.5 and dilutions (50- and 100-fold) were spread on BHI agar containing 10 µg/mL chloramphenicol, 250 µg/mL 5-bromo-4-chloro-3-indolyl-β-D-galactopyranoside (X-gal), and 25 µg/mL fusidic acid. Plates were incubated at 37°C overnight. “White” colonies were picked and streaked on both BHI containing 10 µg/mL chloramphenicol and BHI containing additionally 8 µM hemin (100 mutants per plate). Colonies on the latter plate were stained for catalase activity.

### Inverse PCR

Chromosomal DNA was isolated as described by Marmur [29]. Two µg of the DNA were digested with HindIII or HincII (New England Biolabs) in a 50 µL reaction. Ligation, to obtain circularized DNA, was done after heat inactivation of the restriction enzyme in a total volume of 750 µL containing 2.5 µL T4 DNA ligase (1000 NEB units, 15 Weiss units) and 25 µL (1 µg) digested DNA. The reaction was incubated for 16 hours at 15°C. The DNA was purified and concentrated using the Montage PCR cleanup kit (Millipore). PCR was done using Phusion DNA polymerase (Finnzymes) and primers invCATR2 and invGFPR1 (Table S1) for the EfaMarTn system or primers invISS1fwd and invISS1rev for the IS*S1* system. PCR products were sequenced using primers invISS1fwd or invGFPR1.

### Construction of strain EMB44

A gene deletion cassette consisting of the tetracycline resistance gene *tetL* from pDG1515 [30] flanked by segments of *cydA* and *cydD* from *E. faecalis* OG1RF was constructed using *E. coli* TOP10 as a cloning host (Table 2). The segments were amplified by PCR using primer pair tetL01/tetL02, cydA03/cydA04, and cydD03/cydD04, respectively (Table S1). Vector pLUMB25 was constructed by first cloning the *tetL* PCR product into plasmid pCR-Blunt II TOPO (Invitrogen) and subsequently adding the *cydA* and *cydD* segments via restriction sites introduced by the primers. The c*ydA'*-*tetL*-*cydD'* cassette was transferred from pLUMB25 into the *E. coli*/*E. faecalis* shuttle vector pJRS233 [31] via XhoI and HindIII restriction sites resulting in vector pLUMB28. *E. faecalis* OG1RF was transformed with pLUMB28 by electroporation [28] and transformants were selected at 30°C on TH containing 10 µg/mL erythromycin. Chromosomal integration of the plasmid was selected by incubation at 42°C in the presence of erythromycin and 10 µg/mL tetracycline. Excision of the plasmid by homologous recombination was accomplished by incubation at 30°C in the absence of antibiotics. Clones containing the cassette were selected on tetracycline-containing TH plates incubated at 37°C. The presence of the cassette and the absence of the vector backbone were confirmed by streaking colonies on TH containing tetracycline and erythromycin, respectively. Colonies that were Tet^r^ and Erm^s^ were checked for the loss of the *cyd* locus by colony PCR using primers cyd01 and cyd02 (Table S1).

### *katA* sequence analysis

The *katA* gene in isolated chromosomal DNA was amplified by PCR using primers KatA03 and KatA04 (Table S1). Purified PCR products were sequenced using primers KatA03, KatA04 and KatAR01.

### Large scale cell extracts

Bacteria from a starter culture in hemin supplemented medium (TH or BHI) were used to inoculate 600 mL of the same medium. Cultures were incubated at 37°C and 200 rpm overnight protected from light. Cells were harvested by centrifugation for 20 min at 5,000× *g* and 4°C. The cell pellet was washed once in 50 mM potassium phosphate buffer pH 8.0 and stored at −20°C. For cell lysis the pellet was thawed and suspended in buffer containing 1 mM MgSO_4_, 1 mg/mL lysozyme and some grains of DNase. The cell suspension was incubated for 1 hour at 37°C and subsequently passed two times through a pre-cooled French Press cell operated at 16,000 psi. Unbroken cells and debris were then removed by centrifugation for 20 minutes at 5,000× *g* and 4°C. After centrifugation for 90 min at 200,000× *g* and 4°C the supernatant was saved as cytoplasmic fraction and the pellet (particulate fraction) was washed once in buffer using a homogenizer. The respective fraction contained 5–20 mg/mL protein and were stored at −80°C until analysis.

### Small scale lysates

Fifty mL of TSBG supplemented with hemin were inoculated with bacteria to an OD_600_ of 0.05. The culture was grown for 18 hours at 37°C and 200 rpm. Cells were harvested by centrifugation for 10 min at 8,000× *g* and 4°C. The pellet was washed once in 50 mM potassium phosphate buffer pH 8.0 and was subsequently stored at −20°C. Cells were thawed, suspended in buffer and lysis was done in a FastPrep instrument (MP Biomedicals) at 6 m/s for 3×20 seconds with 0.1 mm zirconia/silica beads. Cell debris and unbroken cells were removed by centrifugation for 30 min at 5,000× *g* and 4°C. Lysates contained about 5 mg/mL protein and were stored at −20°C until analysis.

### SDS-PAGE and Western Blot

SDS-PAGE was done using the NuPAGE system (Invitrogen) with precast 10% Bis-Tris gels and MOPS SDS running buffer. Proteins were then transferred by electroblot onto a PVDF membrane (Millipore). KatA antigen was detected using rabbit KatA antiserum [2] and a HRP-coupled anti-rabbit secondary antibody (GE Healthcare). For detection of bound antibodies the Super Signal West pico kit (Pierce Chem. Co.) and a Kodak Imager station were used.

### Catalase activity

Cell extract (10–50 µL, ≈200 µg total protein) was added to a cuvette containing 0.1% hydrogen peroxide in 3 mL 50 mM potassium phosphate buffer pH 7.0. The rate of hydrogen peroxide decomposition was recorded as the change in absorption at 240 nm. The values were normalized for protein concentration determined using the BCA protein assay (Pierce Chem. Co.).

### Absorption spectroscopy

The presence of cytochrome *bd* was detected by light absorption difference (dithionite-reduced minus air-oxidized) spectroscopy of isolated membranes (2.5–5 mg protein/mL) in 50 mM potassium phosphate buffer pH 8.0. Spectra were recorded at room temperature on a Shimadzu UV 3000 spectrophotometer using a 1 nm slit and 1 mL cuvettes.

### Heme saturation of *oppB*-defective mutant

Strains EMB6 and OG1RF were grown in TSBG until OD_600_ reached 0.2. Then 0.1 µM hemin was added to the cells and incubation continued. Cells were harvested at early stationary phase and KatA polypeptide was detected in cell lysates by immunoblot.

## Supporting Information

Table S1 List of primers.
(PDF)

Table S2 Loci for which a single transposon insertion was found. Colonies of all strains showed decreased catalase activity on hemin-supplemented plates. Catalase activity and KatA content in cell extracts are expressed as fraction relative to those of the parental strain OG1RF.
(PDF)
