# Supplementary material for: Genes Important for Catalase Activity in Enterococcus faecalis
Source: PLoS One. 2012 May 10;7(5):e36725. doi: 10.1371/journal.pone.0036725 (PMC3349705; doi:10.1371/journal.pone.0036725)
Supplement: Table S1 — List of primers. (PDF) [file pone.0036725.s001.pdf]

Table S1. List of primers.

Name	Sequence
**InvCATR2**	5’-GCTCTGCAACTTCATCATTCTG-3’
**InvGFPR1**	5’-CTTCACCCTCTCCACTGACA-3’
**InvISS1fwd**	5’-CGGATTTTCGGTATCTACTGAG-3’
**InvISS1rev**	5’-CAGAAAACTTTGCAACAGAACC-3’
**TetL01**	5’-AAAAGATTAAATTATTGCTTGGTG-3’
**TetL02**	5’-AAAGGATCAATTTTGAACTCTCTC-3’
**CydA03**	5’-CTGCAGCCGATTGAACCGAACAAAGT-3’
**CydA04**	5’-CTCGAGGGGGTTGTAACCGGGATTAT-3’
**CydD03**	5’-AAGCTTGCCACGATCAATTCGGTAAA-3’
**CydD04**	5’-GGATCCGAGGCATTTGGTGAGACGAT-3’
**Cyd01**	5’-TGCTTTAATCAACAACCAGTCG-3’
**Cyd02**	5’-AGGCAAATCAGGGAGGAAAT-3’
**KatA03**	5’-CGCTGAATGACCAATAAAAACG-3’
**KatA04**	5’-CCCAACTAGCTTAGCAAACAAC-3’
**KatAR01**	5’-TCATCGTGCCAACTTCAATC-3’
