# Supplementary material for: Genes Important for Catalase Activity in Enterococcus faecalis
Source: PLoS One. 2012 May 10;7(5):e36725. doi: 10.1371/journal.pone.0036725 (PMC3349705; doi:10.1371/journal.pone.0036725)
Supplement: Table S2 — Loci for which a single transposon insertion was found. Colonies of all strains showed decreased catalase activity on hemin-supplemented plates. Catalase activity and KatA content in cell extracts are expressed as fraction relative to those of the parental strain OG1RF. (PDF) [file pone.0036725.s002.pdf]

Table S2. Loci for which a single transposon insertion was found. Colonies of all strains showed decreased catalase activity on hemin-supplemented plates. Catalase activity and KatA content in cell extracts are expressed as fraction relative to those of the parental strain OG1RF.

**Locus tag**[a]	**Gene**	**Annotated function**	**Strain**	**Tn insertion position in genome**[a]	**Catalase activity**[b]	**KatA protein**
**OG1RF_10055**	*glmU*	UDP-N-acetylglucosamine diphosphorylase	EMB28	59,086	0.53	0.49
**OG1RF_10137**		ABC superfamily ATP binding cassette transporter, ABC protein	EMB42	150,281	0.30	0.34
**OG1RF_10209**	*ftsH*	Cell division protein FtsH	EMB39	207,258	0.31	0.77
**OG1RF_10851**	*galT*	UDP-glucose--hexose-1-phosphate uridylyltransferase	EMB19	892,863	0.17	0.45
**OG1RF_10957**	*rnjB*	Ribonuclease J2	EMB29	998,933	0.64	0.33
**OG1RF_11664**	*cydC*	ABC superfamily ATP binding cassette transporter, ABC/membrane protein	EMB4[c,d]	1,735,103	0.01[d]	0.01[d]
**OG1RF_11691**		FemAB family peptidoglycan biosynthesis protein	EMB9	1,763,492	0.25	0.75
**OG1RF_11704**	*miaA*	tRNA isopentenyltransferase	EMB25	1,782,613	0.08	0.25
**OG1RF_12095**		CBS domain protein	EMB30	2,215,959	0.27	0.11
**OG1RF_12228**	*folC*	Tetrahydrofolate synthase	EMB43[c]	2,346,976	0.27	0.16
**OG1RF_12365**	*rnc2*	Ribonuclease III	EMB38	2,502,158	0.60	0.75
**OG1RF_12384**		Non-specific serine/threonine protein kinase	EMB21[c]	2,523,714	0.28	0.67
**OG1RF_12532**	*mdeA*	Putative methionine gamma-lyase	EMB31	2,682,810	1.16	0.54

[a] GenBank: CP002621.1.
[b] Catalase activity of strain OG1RF was 14 U/mg of protein.
[c] IS*S1* system.
[d] Point mutation in *katA*.
